# Efficacy and Safety of Murivenna Anal Infiltration Compared to Diltiazem Topical Application in Chronic Anal Fissure: Protocol for a Prospective, Randomized, Open-Label Clinical Trial

**DOI:** 10.2196/63063

**Published:** 2025-02-03

**Authors:** Pratap Shankar KM, Indu Puthan Purayil, Palengara Binitha, Amit Kumar Rai, Sophia Jameela, Azeem Ahmad, Chandra Sekhara Rao Bhogavalli, Narayanam Srikanth, Rabinarayan Acharya

**Affiliations:** 1 National Ayurveda Research Institute for Panchakarma Central Council for Research in Ayurvedic Sciences New Delhi India; 2 Ayurvedic and Unani Tibbia College New Delhi India; 3 Central Council for Research in Ayurvedic Sciences New Delhi India

**Keywords:** chronic anal fissure, murivenna anal infiltration, Ayurveda, efficacy, anal infiltration, murivenna, diltiazem, app, chronic, study protocol, randomized clinical trial, proctologic condition, proctologic, pain, quality of life, well-being, surgical intervention, defecation, cessation of bleeding, treatment

## Abstract

**Background:**

Anal fissure is a common proctologic condition that causes significant pain and anguish to patients, significantly impacting their quality of life and well-being. There are various treatment options for anal fissure, ranging from pharmacological agents that reduce anal sphincter tone to surgical interventions for cases resistant to medical management. Ayurvedic treatments have shown potential for the therapeutic management of anal fissure.

**Objective:**

This clinical study aims to analyze the efficacy and safety of murivenna anal infiltration compared to diltiazem topical application in chronic anal fissure.

**Methods:**

This is an open-labeled, randomized, controlled parallel group clinical trial with a sample size of 66 participants to be randomized and allocated in a 1:1 ratio to 2 groups. The intervention group will be treated with murivenna anal infiltration, and the control group will be treated with topical application of diltiazem for a period of 4 weeks. The primary outcome will be the proportion of participants demonstrating complete healing after 4 weeks of treatment. The secondary outcomes will be the proportion of participants demonstrating complete healing after 7 days and 14 days of treatment, change in pain at or after defecation, cessation of bleeding, and any recurrence during the study period. Any adverse events will also be recorded during the trial period.

**Results:**

The project was funded in July 2023, and the study period is 24 months. Participant recruitment started in December 2023. As of August 2024, we have enrolled 50 participants. The data analysis will be complete by June 2025, and the results are expected to be published by August 2025.

**Conclusions:**

High recurrence rates, adverse effects, incomplete healing, and the negative impact on patients’ daily activities and quality of life underscore the need for alternative therapeutic options. Ayurveda offers potential for more sustainable relief with fewer adverse effects. Murivenna oil is a time-tested medicated oil effectively used by Ayurvedic physicians for various ulcers of traumatic and pathological origin. This study will provide scientific evidence on the efficacy and safety of murivenna anal infiltration; further, it can be incorporated into the cost-effective management of chronic anal fissure.

**Trial Registration:**

Clinical Trials Registry India CTRI/2023/09/057330; https://tinyurl.com/y4ut9e8p

**International Registered Report Identifier (IRRID):**

DERR1-10.2196/63063

## Introduction

Anal fissure is a painful tear or split in the distal anal canal. It is a common proctologic condition characterized by intense, prolonged anal pain after defecation, bleeding, and a significant deterioration in the patient’s quality of life. The symptoms of anal fissure are a cause of considerable morbidity to the patient. The condition presents with symptoms including severe pain on defecation, which lasts from a few minutes to several hours, bleeding from the rectum, anal discharge, and swelling. If acute, there may be severe pain; the intensity will be comparatively less in the chronic phase. Spontaneous healing of anal fissures is rare due to the reactive spastic contraction of the internal anal sphincter, which diminishes blood flow to the affected area. This constriction impedes the natural healing process, prolonging the duration of symptoms such as pain and bleeding. Therapeutic intervention is often required to alleviate discomfort and promote healing. Various treatment modalities aim to relax the anal sphincter, increase blood flow, and facilitate tissue repair, thereby addressing the underlying cause and promoting resolution of the fissure. Conservative management includes, among other approaches, bulk agents, stool softeners, warm sitz baths, botulinum toxin injections, or topical application of ointments like diltiazem and glyceryl nitrate [1]. New treatment options, such as hyperbaric oxygen therapy to increase tissue oxygenation and induce wound healing, are now being studied [2]. Considerable drawbacks are reported, such as recurrences, toxicity, headaches, and giddiness, while using these external ointments [3]. Surgical treatment is adopted for those cases in which nonsurgical treatment for more than 6 to 8 weeks does not produce desirable results or when there is a recurrence of anal fissure. Surgical treatments include Lord dilatation, lateral internal sphincterotomy, advancement flaps, and fissurectomy [4,5]. Complications of surgical treatments include incontinence to flatus and feces, nonhealing external wounds, abscess, and fistula formation [6]. Miscellaneous novel therapies such as sacral nerve stimulation, autologous adipose tissue transplantation, and posterior tibial nerve stimulation are now being examined as alternatives to lateral sphincterotomy and reliable procedures to avoid fecal incontinence [7]. However, pharmacological modalities are more preferred for treatment of anal fissure, as they are well tolerated, with minimal to no side effects. *Susrutha Samhitha*, a comprehensive Ayurvedic textbook on surgical and parasurgical practices of ancient India, mentions anal fissures as being iatrogenic, and mentions a condition called *parikartika* (“cutting pain in the anus”) in the context of *vaidya nimitha bastivyapat* (“indiscretion of the clinician while administering a medicated enema”) and *gudakshata* (“ulceration/injury in the anus”) in *bastinetravyapat* (“complications due to the enema pipe”). The book further says that this condition should be treated in the same way as traumatic wounds [8]. The *Susrutha* advocates oil irrigation or infiltration to pacify *pitta dosa* (metabolic and biochemical processes that generate heat), which is considered pivotal in the pathology of inflammation and wound formation. [8] *Murivenna* oil is a time-tested medicated oil that treats various exogenous and endogenous ulcers. The drugs used for the preparation of murivenna oil, namely *Aloe vera*, *Pongamia glabra*, *Borreria hispida*, *Asparagus racemosus*, and *Moringa oleifera* are *pittasamaka* (pacifying *pitta*) and *vranavasadana* (ie, they reduce the hypergranulation of wounds). Further, they have been proven to have analgesic and anti-inflammatory properties [9]. Murivenna can potentially help to lessen pain and spasm and promote healing of anal fissures. Further, the anal infiltration process enables the medicine to be retained in the anal canal for an extended period, which can help in reducing increased sphincter tone and associated symptoms.

Hence, this clinical study will compare the effect of murivenna anal infiltration against diltiazem topical application in the treatment of chronic anal fissures. The primary objective of the study is to determine the efficacy of murivenna anal infiltration for healing of anal fissures in comparison with a topical application of 2% diltiazem. The secondary objectives are to determine the safety and efficacy of murivenna anal infiltration for the reduction of pain and bleeding and the prevention of recurrences of anal fissure in comparison with the topical application of 2% diltiazem.

## Methods

### Study Design and Setting

The study is an open-label, randomized, controlled, parallel group clinical trial conducted at the National Ayurveda Research Institute for Panchakarma (NARIP), Cheruthuruthy, Thrissur District, Kerala, India. The schedule of enrollment, intervention, assessments, and follow-up visits for the study participants is given in Table 1.

**Table 1:** Schedule of screening, enrollment, intervention assessments, and follow-up in the clinical trial.

Content	Screening	Intervention period						Follow-up period				
		Day 1 (baseline)	Day 8	Day 15	Day 22	Day 30 (treatment end)	Day 60		Day 90	Telephonic follow-up				
										Month 1	Month 2	Month 3
Eligibility evaluation	✓											
Provision of participant information sheet	✓											
Informed consent	✓											
Medical history and demographic profile		✓										
Clinical examination		✓	✓	✓	✓	✓	✓		✓			
Assessment of subjective parameters		✓	✓	✓	✓	✓	✓		✓	✓	✓	✓
Laboratory test	✓					✓						
Drug compliance assessment			✓	✓	✓	✓						
Rescue medication assessment			✓	✓	✓	✓	✓		✓	✓	✓	✓
Adverse events assessment			✓	✓	✓	✓						
Recurrence of disease							✓		✓	✓	✓	✓

### Study Participants

#### Inclusion Criteria

Participants of either sex will be included if they are in the age group of 16-65 years, have a chronic anal fissure persisting for more than 6 weeks, are capable of and freely willing to provide written informed consent prior to participation in the study, and comply with the study protocol requirements.

#### Exclusion Criteria

Individuals with comorbidities, including uncontrolled diabetes mellitus, hypertension, anemia, malnourishment caused by systemic disease, fistula in ano, hemorrhoids, perianal abscess, clinically evident fecal incontinence and anal stenosis/fibrosis, inflammatory bowel disease, tuberculous ulcer, malignancies, HIV, clinically significant renal disease, hepatic disease and cardiovascular disease, psychological disease (eg, anxiety and depression), and active substance abuse will be excluded; participants using medications hampering wound healing will also be excluded. Patients with fissures associated with abscess, drug-induced fissures, fissures resulting from external trauma, fissures located at lateral locations, and multiple fissures will be excluded. Participants using oral calcium channel blockers, having sensitivity to the intervention drugs or calcium channel blockers, with frequent history of headaches, or using drugs such as steroids or nonsteroidal anti-inflammatory drugs, either for fissure or any other unrelated disease or condition, will be excluded from the study. Pregnant and lactating women and participants who have used either of the trial interventions within 30 days prior to the trial’s randomization will be excluded from the trial.

### Study Intervention

Participants in the intervention group will be treated with anal infiltration of murivenna oil for 4 weeks. The medical team will administer the oil through the anal canal using sterilized rubber tubes attached to syringes at baseline. Either the participant or the attendee will receive education on the procedure and will be asked to perform the procedure on subsequent days until the next visit. The participant should lie down for 15 minutes after completing the procedure. In the first week, the patients will receive anal infiltration of 30 ml of murivenna oil once daily, and for the remaining 3 weeks, they will receive anal infiltration of 20 ml of murivenna oil once daily. During the trial period, participants in the intervention group will receive *triphala choornam* (another Ayurvedic medicine; 10 g) at bedtime as a mild laxative, and the participants will be further advised to perform a sitz bath with triphala kashayam once a day. Participants in the control group will be treated with 2% diltiazem gel for 4 weeks. They will be instructed to apply the gel at least 1.5 cm to 2 cm into the anus in the morning and at night after the sitz bath. During the trial period, participants in the control group will receive lactulose syrup (15 ml) at bedtime as a laxative. Participants in both groups will be advised to consume foods rich in dietary fiber and avoid activities that could cause microtrauma to the anus, such as prolonged sitting or traveling on a bicycle or motorcycle.

The murivenna oil was manufactured at the Good Manufacturing Practices (GMP)–certified pharmacy at NARIP, and the triphala choornam was manufactured by a GMP-certified pharmacy at the Central Ayurveda Research Institute, Jhansi, India, as per the respective standards available in the Ayurvedic Pharmacopoeia of India [10]. Table 2 lists the ingredients of murivenna oil.

**Table 2:** Ingredients of murivenna oil.

Serial number	Ingredient	Sanskrit name	Quantity	Part used
1	*Pongamia glabra* Vent	Karanja	384 g	Bark
2	*Piper betle* L	Tambuli	384 g	Leaf
3	*Aloe vera* L	Ghritakumar	384 g	Leaf
4	*Erythrina indica* Lam	Mura	384 g	Leaf
5	*Allium cepa* L	Palandu	384 g	Bulb
6	*Moringa oleifera* Lam	Sobhanjana	384 g	Leaf
7	*Borreria hispida* (L) Schum	Madanaghanti	384 g	Whole Plant
8	*Asparagus racemosus* Wild	Shatavari	192 g	Rhizome
9	Coconut oil	—^a^	768 ml	Oil

^a^Not applicable.

### Outcome Measures

The primary outcome will be the proportion of participants who undergo complete healing after 4 weeks of treatment. The secondary outcome measures will include the proportion of participants with complete healing after 7 days and 14 days of treatment, respectively. In the present context, we define healing of the fissures as the disappearance of symptoms and the evidence of fissure re-epithelization, which the investigators will record based on an examination. The investigators will categorize the healing as follows at each follow-up visit: none, partial, or complete.

Another secondary outcome measure will be the mean change in pain intensity during or after defecation. The investigators will ask the participants to record their pain intensity during or after defecation over the last 24 hours using a 100-mm visual analog scale at each follow-up. The investigators will also record how long it takes for participants to experience an improvement in pain intensity and cessation of bleeding, as well as how many participants require analgesics for pain relief. The investigators will advise the participants to log their pain intensity, bleeding status, and any need for analgesics in diary log sheets. The team will issue these sheets on the baseline day, as well as on the 8th, 15th, and 22nd days. Investigators will educate the participants on how to fill out the sheets. The participants will be followed up for 5 months after the intervention period to record any recurrence of anal fissure.

### Safety Outcomes

Participant-reported adverse events (AEs) during the trial period will be recorded on every scheduled follow-up visit in a structured format. All AEs during the study will be monitored and appropriate care will be provided. There have been no past reported AEs for murivenna anal infiltration [11]. The predictable AEs for triphala choornam include loose stools, obstipation, change in the sense of taste, nausea, skin lesions, and tiredness [12]. Dizziness, headache, weakness, nausea, or swelling of the hands or feet may rarely occur with the use of diltiazem ointment [13]. Diarrhea, bloating, nausea, vomiting, and stomach pain are reported side effects of lactulose syrup. Rarely reported serious AEs include weakness and irregular heartbeat [14].

### Withdrawal Criteria

Participants not willing to continue or who are noncompliant with the study procedures (a minimum 80% compliance is essential to continue in the study) will be withdrawn from the study. Participants developing life-threatening complications or any other severe illness because of another pathology that requires urgent treatment will also be withdrawn from the study. Participants developing serious AEs or treatment-induced AEs requiring hospitalization will be withdrawn from the study. It will be ensured that these participants receive appropriate incidental care or are referred to a higher medical facility if required. The reasons for withdrawal will be recorded in the participant’s case record form (CRF). The sponsor and the ethics committee will be informed within 2 working days, and will be provided with proper justification.

### Sample Size

The sample size was calculated based on the previous differences in the proportion of participants having complete healing of anal fissures between study groups. In a previously published study, 25% of participants had complete healing of anal fissure by the fourth week of treatment with 2% diltiazem gel, and based on the results of another previously published observational study, we assumed that fissures would heal in nearly 60% of participants treated with the trial intervention [11,15]. A sample size of 30 per group is needed to achieve 80% power with a 95% CI. Adding an attrition rate of 10% results in a sample size per group of 33. Therefore, a total of 66 participants will be enrolled in the trial.

### Recruitment

Participants from the outpatient department of NARIP diagnosed with chronic anal fissure will be screened for their eligibility to participate in the clinical trial. Informed consent will be obtained from the participants before screening. Participants eligible as per the inclusion and exclusion criteria will be allocated to one of the study groups based on the randomization schedule.

### Randomization and Allocation Concealment

A computer-generated randomization number sequence with block randomization will be created by the study statistician independently of the investigators undertaking recruitment and subsequent visits. The statistician will use Stata (version 16; StataCorp) to generate a random sequence, ensuring that each participant is randomly assigned to one of the study arms in a 1:1 ratio. The assignments will be enclosed in sequentially numbered, opaque, sealed envelopes, which will be opened by the participants at the time of enrollment.

### Compliance

Compliance with the prescribed medicines will be monitored through a compliance assessment form issued to the participants on each visit. The participants will be instructed to complete the assessment form after each medicine intake or administration. In addition, compliance will be evaluated by counting the number of containers and tubes returned and the approximate quantity of medications used by each participant. A minimum of 80% compliance is essential for the participant to continue the study.

### Concomitant and Rescue Medication

The participants will be instructed to inform the investigators before taking any type of medication apart from the trial drugs. The investigators will record the details of the medications and the reason for taking them in the CRF. If there is any medical emergency, the use of any rescue medication will be permitted, and this medication will be recorded in the relevant section of the CRF.

### Data Collection and Documentation

Before conducting this clinical study, the investigators and research team will be uniformly trained on Good Clinical Practice (GCP) protocols, trial-specific processes, and documentation. The research team will collect the information and fill in the details in the CRF (Table 1 provides details) for each visit. All documented data will be checked regularly by the principal investigator (PI) to avoid mistakes and omissions. Any modifications made will be clearly visible, and the corrections will be signed and dated by the PI. The data will subsequently be recorded in an e-format and verified as required; the original CRFs will be archived in order with a search catalog.

### Statistical Analysis

All statistical data analyses will be performed using SPSS (version 26.0; IBM Corp). Categorical data will be presented as numbers (percentages) and will be compared using the *χ*^2^ test or Fisher exact test. Continuous variables will be described with either means and SDs for data with a normal distribution or median and IQR for nonnormally distributed data. The within-group analysis will be done using a 2-tailed paired sample *t* test for normal data, whereas the Wilcoxon signed-rank test will be used to compare nonnormal data. Comparisons between the experimental and control groups at each time point will be done using an independent sample 2-tailed unpaired *t* test or Mann-Whitney test for normal and nonnormal data, respectively. *P*≤.05 will be considered statistically significant.

### Monitoring

The monitoring committee set up by the sponsor will conduct on-site or virtual monitoring to ensure adherence to the trial protocol and compliance to GCP and the Central Council for Research in Ayurvedic Sciences Research Policy.

### Trial Audit

The regulatory authorities, the institutional ethics committee (IEC), or the funding agency will audit the trial, and the investigators will ensure access to all the documents related to the study for the on-site audit.

### Ethical Considerations

The study has been approved by the IEC of NARIP (8/16/23/NARIP/Tech meeting/2511; March 31, 2023) and has been registered prospectively at Clinical Trials Registry - India (CTRI) (CTRI/2023/09/057330). The study will be conducted in accordance with the Indian Council for Medical Research National Ethical Guidelines for Biomedical and Health Research on Human Participants (2017). Written informed consent (in English and Malayalam) will be obtained from the eligible participants before screening. All protocol modifications will be communicated to the IEC and funding agency and corrected accordingly in the CTRI. The CRFs will be stored in a secure area, and the participants’ data will be coded to ensure confidentiality. The study participants will be given routine medical care, if required, after the completion of the study period. The study participants will be compensated for any financial losses (eg, loss of wages) with an incidental support of Rs 300 (US $3.49) for each visit.

## Results

The project was funded in July 2023, and the study period is 24 months. The participant recruitment was started on December 2023. As of August 2024, we have enrolled 50 participants. The data analysis will be complete by June 2025, and the results are expected to be published by August 2025. The study outcomes will be disseminated through research articles in peer-reviewed scientific journals and presentations at national conferences.

## Discussion

This randomized, open-label clinical trial is expected to assess the effect of murivenna anal infiltration compared to topical application of diltiazem in the treatment of chronic anal fissure.

### Strength

This is the first randomized clinical trial to evaluate the efficacy of murivenna anal infiltration for chronic anal fissure. The quantitative outcome measures included in this trial will yield valid data for clinical use of murivenna for the treatment of anal fissure.

### Limitations

The study is being conducted at a single center, and hence participants from only a single area are included in the trial. The study will apply to countries where Ayurveda is practiced. However, the procedure can be performed by trained persons of other countries provided they have regulatory permission to use murivenna and triphala choornam. Further, single-pack administrations with a fixed dose of murivenna may be considered for better patient adherence.

### Conclusion

If this clinical trial proves effective, medical professionals can consider murivenna anal infiltration as a potential alternative therapeutic option, which may further be incorporated into cost-effective management for chronic anal fissure.

## Appendix

Multimedia Appendix 1 SPIRIT (Standard Protocol Items: Recommendations for Interventional Trials) checklist.

Multimedia Appendix 2 Consent form and patient information sheet.

## Abbreviations

AE: adverse event
CRF: case record form
CTRI: Clinical Trial Registry - India
GCP: Good Clinical Practice
GMP: Good Manufacturing Practices
IEC: institutional ethics committee
NARIP: National Ayurveda Research Institute for Panchakarma
PI: principal investigator
