# Supplementary material for: Efficacy and Safety of Murivenna Anal Infiltration Compared to Diltiazem Topical Application in Chronic Anal Fissure: Protocol for a Prospective, Randomized, Open-Label Clinical Trial
Source: JMIR Res Protoc. 2025 Feb 3;14:e63063. doi: 10.2196/63063 (PMC11833261; doi:10.2196/63063)
Supplement: Multimedia Appendix 2 [file resprot_v14i1e63063_app2.docx]

**Supplementary file 2: Consent form and patient information sheet**

**PATIENT INFORMATION SHEET**

1. **Study Title:**

“Efficacy and safety of *Murivenna* anal infiltration compared to Diltiazem topical application in chronic anal fissure: A prospective randomized open-label clinical trial”

1. **Invitation**

You are invited to take part in a research study. Before you decide it is important for you to understand why the research is being done and what it will involve, please take time to read the following information carefully and discuss it with friends and relatives if you wish. Ask us if there is anything that is not clear or if you would like more information. Take time to decide whether or not you wish to take part.

1. **What is the purpose of the study?**

This is a research study being conducted under the Intra Mural Clinical Research Programme of the Central Council for Research in Ayurvedic Sciences (CCRAS), Ministry of Ayush, Government of India, involving administration of *Murivenna* anal infiltration in cases of chronic anal fissure with a view to scientifically document the clinical efficacy and safety of the said Ayurvedic formulation which comprises of drugs which have been used since hundreds of years for the treatment of exogenous and endogenous types of ulcers, inflammatory swellings, sprains and fractures.

1. **Why have I been chosen?**

Being a subject of chronic anal fissure, you are considered as an ideal candidate for the study.

1. **Do I have to take part?**

It is up to you to decide whether or not to take part. If you do decide to take part you will be given this information sheet to keep and be asked to sign a consent form. If you agree to take part you are still free to withdraw at any time and without giving any reason. This will not affect the standard of care you receive.

1. **What will happen to me if I take part?**

After obtaining your consent, you will be screened to assess your eligibility to participate in the study. If found eligible, you will receive either (1) Ayurvedic formulations or (2) standard modern medicines. What combination you will get will be determined by computer based randomization and it is but a chance that you may receive either combination- Ayurvedic formulations or standard care treatment mentioned above. If you are getting randomly allocated into study group, you will be prescribed to do anal infiltration with *Murivena*, sitz bath with *Triphala kashyam* and internally to take *Triphala choornam* (10 gm at bed time). If your are allocated into control group, you will be prescribed Ditiazem gel 2% for anal application, sitz bath with warm water and oral intake of syrup Lactulose 15 ml at bed time. A drug compliance form will be given to record the usage of medicines. Irrespective of the allocated study group, you have to consume foods rich in dietary fibre and avoid activities which will cause micro trauma to anus such as prolong sitting, travelling in two-wheelers etc. during the study period. If you agree to take part in this study, you have to undergo general physical examination and laboratory investigations from time to time for the assessment of the effect of the prescribed interventions you would be taking at that time. Investigations will include blood tests. In the first visit i.e. on baseline (Day 1) you may have to devote approximately one hour to facilitate your Investigator in recording your medical history in detail. Thereafter, in the subsequent visits it may take nearly 15-20 minutes to make the assessment. You have to come on every 7^th^ day for follow-up clinical examination and for collecting the medicines for the next 7 days.You have to bring back the containers and drug compliance form filled by you during the next visit. After 4 weeks, blood investigation will be repeated. Later you will be instructed to come for a follow up visit after 30 days (60^th^ day) and after 60 days (90^th^ day) for clinical examination and assessment of subjective parameters. After 90^th^ day, you will be contacted over telephone each month up to three months for assessment of subjective parameters. You will be nominally compensated for your loss of wages/conveyance charges by paying an amount of Rs.300 (Three hundred only) for every visit to the hospital during the study period i.e. Baseline, 8th Day, 15th Day, 22nd Day, 30th Day, 60th Day, and 90th Day, irrespective of study or control group. You are free to contact the Principal investigator of the study at any time to discuss about any type of health issue faced by you during the study period.

1. **What do I have to do?**

You have to adhere to the instructions by the Investigating physician regarding taking the medicines as advised and reporting for follow up on the prescribed day. You should follow and obey the instructions of the investigator very religiously while continuing with the trial drugs. During the course of the trial, you can safely continue with your regular medication, if any (for which you need to intimate your Investigator).

1. **What is the drug or procedure that is being tested?**

| **Arm** | **Intervention** |
| --- | --- |
| **Group A** | ***Murivenna* oil**   - **Dose & Duration:** 30 ml once daily for 1 week followed by 20 ml once daily for three weeks - **Method of administration:** Anal infiltration. Patient’s carer will be taught to infiltrate oil or may be asked to come to OPD if patient’s carer is unavailable or unable to administer. - **Duration of therapy:** 4 weeks   ***Triphala choornam*** 10 gm bed time  Sitz bath with *Triphala kashayam* |
| **Group B** | **Diltiazem gel 2 %**   - **Method of administration:** Apply the gel at least 1.5 cm to 2 cm into the anus - **Time of administration:** Morning and night after sitz bath - **Duration of therapy:** 4weeks Lactulose syrup 15 ml bed time   Sitz bath |
| Patients in both groups will be advised to consume foods rich in dietary fibre and avoid activities which will cause micro trauma to anus such as prolong sitting, travelling in two-wheelers etc | |

1. **What are the alternatives for treatment?**

The alternative treatment options include conventional surgical management.

1. **What are the expected side effects / risks of the treatment?**

The Ayurvedic formulations, *Murivenna* and *Triphala choornam* are in use since ancient times, yet individual specific side effects may appear during the course of the clinical trial, which you are to report immediately to the investigating physician and appropriate treatment will be advised to you. Diltiazem ointment external application may cause local irritation, itching and burning. Rare side effects are headache, nausea & dizziness. Lactulose syrup may cause abdominal distention & bloating and diarrhea. Rare side effects are nausea, vomiting and stomach ache

1. **What are the possible benefits of taking part?**

The expected benefits of the study to you will be healing of anal fissure, reduction of anal pain and anal bleeding, but it is not guaranteed that you will definitely get benefits after completing the course of the study intervention. Your participation will help us in generating sufficient data to validate the efficacy and safety of Ayurveda formulations in managing chronic anal fissure.

1. **What if new information becomes available?**

If during the course of the clinical trial, some new information becomes available about the trial interventions, you will be informed about that by your investigating physician after which you are free to decide whether you want to continue in the study or not. If you decide to withdraw, this will not at all affect your routine care in the hospital. If you decide to continue in the study, you will be asked to sign a fresh consent form. On the other hand, upon receiving any new information, the investigating physician might consider it to be in your best interests to withdraw you from the study. Your investigating physician will explain the reasons for dropping you from the study and arrange for the routine care to continue.

1. **What happens when the research study stops?**

You will be given appropriate advice for future line of treatment.

1. **What if something goes wrong?**

Compensation for any adverse effect caused by taking part in this study and related to study interventions would be taken care by appropriate insurance coverage, treatment or referrals.

1. **Will my taking part in this study be kept confidential?**

Yes, the patient information will be kept confidential but any of the medical records may be inspected by the sponsors for the purpose of analyzing the results. They may also be looked at by members of Institutional Ethics Committee and by Regulatory authorities / Court to check that the study is being carried out correctly. Your name, however, will not be made public and any sensitive matter regarding the state of health will be kept confidential.

1. **What will happen to the results of the research study?**

The results of the clinical trial will be published in indexed medical journals so that other doctors and researchers can benefit from the results. You can ask the investigating physician for a copy of the publication. If published, the identity and personal details of the patient will be kept strictly confidential. No named information about you will be published in any of the trial reports.

1. **Who is organizing and funding the research?**

Central Council for Research in Ayurvedic Sciences (CCRAS), New Delhi is sponsoring the research. National Ayurveda Research Institute for Panchkarma, Cheruthuruthy is organizing and coordinating the study.

1. **Who has ethically reviewed the study?**

This study has been ethically approved by an independent body known as Institutional Ethics Committee of National Ayurveda Research Institute for Panchkarma, Cheruthuruthy comprising of medical experts, non-experts and members of the general public.

1. **Contact for further information**

If desirous of any relevant information at any stage of the clinical trial, you may feel free to ask the investigating physician for that information. You would be given a copy of the information sheet and a signed consent form.

**Contact address of the Principal Investigator:**

Dr. K.M. Pratap Shankar

Research Officer (Ay.), NARIP, Cheruthuruthy, Thrissur Dt, Kerala 679531

Mobile No. 9744824014/9061227458; E-mail: [kmpvarma@gmail.com](mailto:kmpvarma@gmail.com)

**Contact on behalf of Sponsor:**

Director, National Ayurveda Research Institute for Panchkarma, Cheruthuruthy

Thrissur Dt, Kerala 679531

Phone No. – 04884 262544

Email – nrip.cheruthuruthy@gmail.com

**Efficacy and safety of *Murivenna* anal infiltration compared to Diltiazem topical application in chronic anal fissure: A prospective randomized open-label clinical trial**

**CONSENT FORM—TO BE SIGNED BEFORE SCREENING**

I __________________________________________, confirm that I have read the information sheet for the study titled “Efficacy and safety of Murivenna anal infiltration compared to Diltiazem topical application in chronic anal fissure: A prospective randomized open-label clinical trial”. I had the opportunity to consider the information, ask questions and got them answered satisfactorily. I understand that my participation is voluntary and that I am free to withdraw at any time without giving any reason, without my medical care or legal rights being affected. I understand that the Sponsor or monitoring committee may review relevant data collected during the study, and I give permission for these individuals to have access to my records. I do hereby, exercising my free power of choice, give my consent to be included as a subject in this research study, which is being executed at National Ayurveda Research Institute for Panchkarma, Cheruthuruthy.

**Name of the Participant Signature Date**

**Name of the Witness Signature Date**

**(in case of illiterate subject)**

**Name of the Principal Investigator Signature Date**

**NB: - Two copies should be signed, one to be retained in the participant file by the investigator and another to be provided to the participant.**
